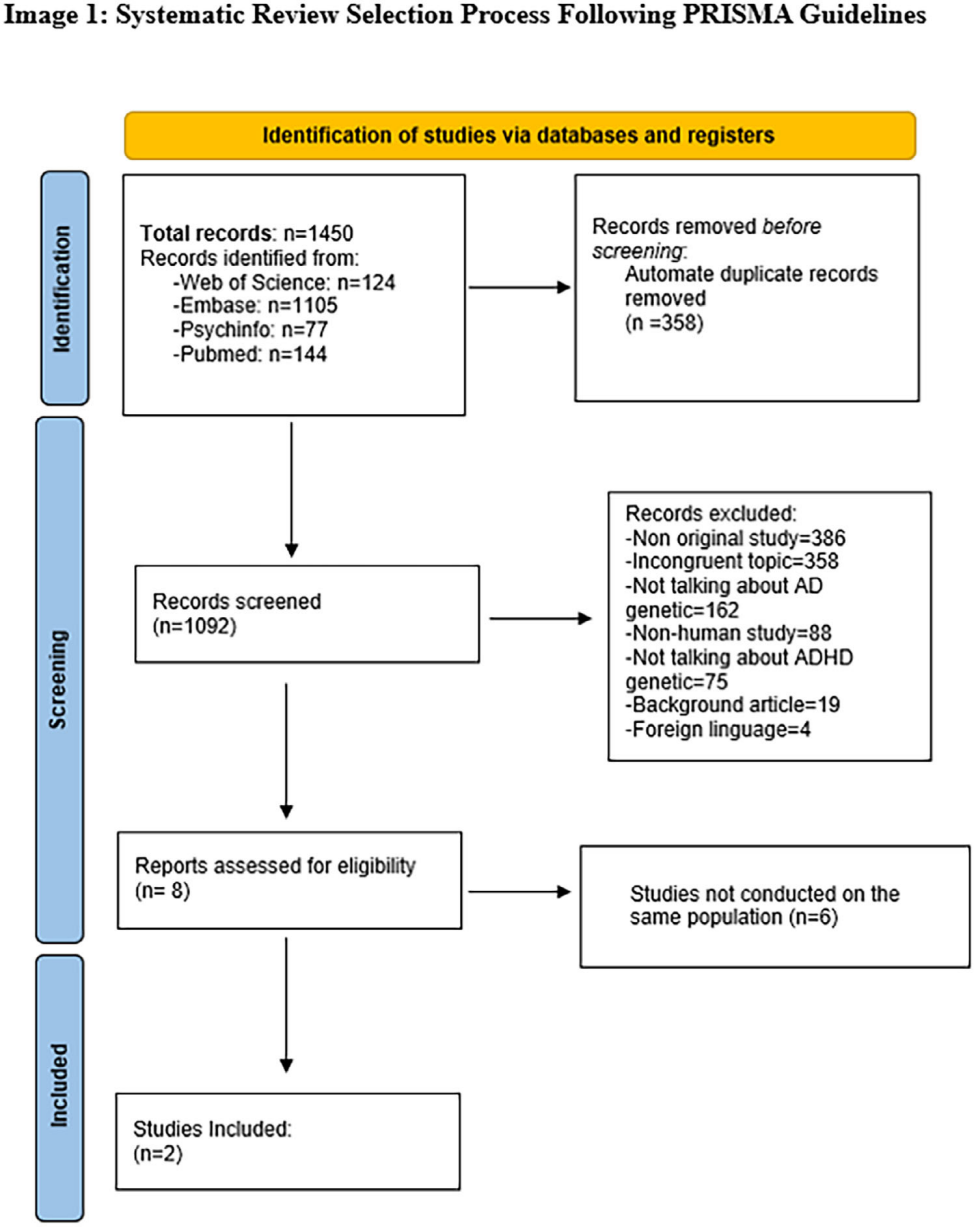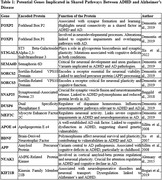# Genetic Intersections of ADHD and Alzheimer's Disease: A Systematic Review

**DOI:** 10.1002/alz70857_101881

**Published:** 2025-12-24

**Authors:** Riccardo Borgonovo, Martino Ceroni, Lisa Marta Arnaud, Lucia Morellini, Marianna Lissi, Leonardo Sacco

**Affiliations:** ^1^ Neurocenter of Southern Switzerland, Lugano, Ticino, Switzerland

## Abstract

**Background:**

Attention‐deficit/hyperactivity disorder (ADHD) and Alzheimer's disease (AD) are distinct neurological conditions with emerging evidence of shared genetic and molecular mechanisms. ADHD, affecting 5% of children and 3‐4% of adults worldwide, is characterized by inattention, hyperactivity, and impulsivity. AD, the leading cause of dementia, involves progressive neurodegeneration marked by amyloid‐beta (Aβ) plaques and tau tangles. Shared pathways, including synaptic plasticity, neuroinflammation, and oxidative stress, may link these disorders.

**Methods:**

A systematic review adhering to PRISMA guidelines was conducted to investigate potential genetic overlaps between ADHD and AD. Searches in PubMed, Embase, PsychInfo, and Web of Science identified studies using genetic analyses, particularly ADHD polygenic risk scores (PRS). Studies involving human participants with confirmed AD diagnoses were included. Screening and selection processes used Rayyan software. The review was conducted between October 2023 and December 2024.

**Results:**

Two studies met inclusion criteria. Leffa et al. (2023) found that ADHD‐PRS correlated with progressive cognitive decline, tau pathology, and frontoparietal atrophy in Aβ‐positive individuals. Garcia‐Argibay et al. (2022) associated ADHD‐PRS with increased risks for cardiometabolic and autoimmune conditions but found no direct link to dementia. Key genes implicated in both conditions include FOXP2, APOE ε4, and SNAP25, involved in synaptic plasticity and neuronal viability.

**Discussion:**

Findings suggest genetic liability for ADHD may exacerbate AD pathology, particularly in the presence of Aβ. Shared pathways like Wnt/mTOR signaling and neuroinflammation underscore potential intervention targets. However, conflicting evidence highlights the need for longitudinal studies tracking ADHD populations to clarify causal relationships.

**Conclusion:**

This review identifies genetic and molecular overlaps between ADHD and AD, emphasizing the potential for early identification of at‐risk populations through PRS. Future research should prioritize longitudinal studies and interventions targeting shared pathways to mitigate neurodevelopmental and neurodegenerative risks.

**Bibliography**

Garcia‐Argibay, M., du‐Rietz, E., Lu, Y., Martin, J., Haan, E., Lehto, K., Bergen, S.E., Lichtenstein, P., Larsson, H., & Brikell, I. (2022). The role of ADHD genetic risk in mid‐to‐late life somatic health conditions. *Translational Psychiatry, 12*(1), 152. https://doi.org/10.1038/s41398‐022‐01919‐9.

Leffa, D.T., Gilsanz, A.H., Solomon, I.H., Salinas, J., Laibson, M.J., & Bandeen‐Roche, K.A. (2023). ADHD polygenic risk scores influence Alzheimer's disease pathology: A longitudinal biomarker study. *Neurobiology of Aging*. https://doi.org/10.1016/j.neurobiolaging.2023.03.015.